# Is it really the result of a concussion? Lessons from a case study

**DOI:** 10.1186/s40798-019-0181-4

**Published:** 2019-03-04

**Authors:** Joshua P. McGeown, Patria A. Hume, Stephen Kara, J. Patrick Neary, Will Gardner

**Affiliations:** 1 Sports Performance Research Institute New Zealand (SPRINZ), Faculty of Health and Environmental Science, Auckland University of Technology, Private Bag 92006, Auckland, 1142 New Zealand; 2 National Institute of Stroke and Applied Neuroscience (NISAN), Faculty of Health and Environmental Science, Auckland University of Technology, Auckland, New Zealand; 3 Axis Sports Concussion Clinic, Auckland, New Zealand; 4 Faculty of Kinesiology and Health Studies, University of Regina, Regina, SK Canada; 5 Auckland, New Zealand

**Keywords:** Sports medicine, Concussion, Vestibular, Assessment, Persistent symptoms

## Abstract

**Background:**

Within the last two decades, attitudes have shifted from considering sports-related concussion as an insignificant minor injury with no long-term repercussions to a potentially serious brain injury garnering attention from media, clinicians, researchers, and the general public.

**Objectives:**

To conduct a case study to determine the underlying cause of persistent issues suspected to be associated with a history of sports-related concussion.

**Protocol:**

Participant A underwent neurophysiological testing following the Neary protocol (assessment of cerebrovascular and cardiovascular variables), comprehensive concussion assessment at a dedicated sports concussion clinic (history, neurological assessment, cervical spine screening, vestibulo-ocular screening, SCAT-5, and exercise testing), referral to a neurologist, structural MRI scan, and referral for specialised assessment at a dedicated dizziness and balance centre.

**Results:**

Despite a history of multiple sports-related concussions, Participant A’s persistent symptom reports were associated with peripheral vestibular dysfunction and otolithic dysfunction seemingly unrelated to his concussion history.

**Discussion:**

Lessons from Participant A’s case study showed that on-going symptoms that patients may associate with the effects of concussions may instead be due to unrelated causes that share similar symptomology.

**Conclusion:**

This research exemplifies the importance of a multi-disciplinary assessment using a repeated testing protocol.

## Key points

- A major challenge of managing patients with a history of sports-related concussion (SRC) is the comorbidity shared between symptoms of SRC and an extensive array of other potentially unrelated conditions.
- Findings of this case study show that on-going symptoms that patients may associate with the effects of concussions may not be related to concussion. Caution is necessary to confirm that all possibilities for contributing factors have been considered for SRC symptoms that are comorbid in nature with a variety of other conditions.

## Introduction

In recent years, the acute and long-term effects of a sports-related concussion (SRC) have garnered increased concern and attention from researchers, medical practitioners, media reporters, athletic organisations administrators, and the general public. Within the last two decades, attitudes have shifted from considering SRC as an insignificant minor injury with no long-term repercussions to a potentially serious brain injury. Changes in perceptions have been driven by rapidly evolving evidence within the literature regarding SRC epidemiology, underlying mechanisms of SRC, symptoms, assessment, rehabilitation/return-to-play, and potential long-term repercussions of a history of SRC [1]. This evolving evidence has facilitated substantial rule changes in a variety of sporting codes to decrease the likelihood of an athlete sustaining an impact that may result in SRC and persistent concussion symptoms (PCS). For example, New Zealand’s Accident Compensation Corporation (ACC) has worked together with Auckland University of Technology’s (AUT) Sport Performance Research Institute New Zealand (SPRINZ), NZ Rugby Union, NZ Rugby League, NZ Football, and NZ Netball staff to develop and release national guidelines for sport concussions as part of the ACC SportSmart initiative [2]. The intent of these national guidelines is to standardise how to recognise SRC, remove athletes from play, refer the athlete to proper medical attention, and how to appropriately return athletes to school/work and subsequent reintegration to sport and activity following SRC [2].

World Rugby, NZ Rugby, and AUT conducted the inaugural NZ Rugby Health project to explore the longer-term impacts of playing rugby on general health and cognitive function. Since the initiation of the NZ RugbyHealth project the investigation into the effects on general health and cognitive function from playing rugby has expanded into a Global Rugby Health Research Programme (GRHRP). This GRHRP involves collaboration between researchers in New Zealand, the United Kingdom, Canada, Australia, and the United States of America, to better understand the long-term health of retired rugby players. Published findings [3] from the NZ RugbyHealth project generated considerable media attention worldwide resulting in an increased awareness within the general public of the potential effects of a history of SRC sustained from participating in different rugby codes [4, 5]. This media attention led Participant A, aged 40, to contact NZ RugbyHealth project principal investigator Professor Patria Hume of AUT. Participant A shared his history of 10 self-reported SRCs that he sustained from rugby, skiing, and mountain biking, and explained that he felt as though he was suffering from the long-term consequences of his SRCs on a regular basis, and thus possibly experiencing PCS [6]. Participant A described a history of symptoms he had been experiencing over the last 8 years including: pressure headaches, nausea without vomiting, feeling foggy, dizziness, feeling as if the room was spinning/tilting, and sporadic severe ‘head spins’ lasting 15–60 s that would leave him feeling disoriented. Furthermore, Participant A noted that sleep, stress reduction, hydration, and quality nutrition aided in improving these spontaneous incidents of dizziness and disorientation. Prior to learning about the RugbyHealth Project, Participant A had made efforts to try and determine the cause of the problems he was experiencing by organising referral for a structural MRI and consultations with an otolaryngologist, respiratory physician, and orthopaedic surgeon. The findings from the MRI and specialist consultations all came back negative. Participant A was informed he was in good health and did not receive any explanation for the symptoms he was suffering. Feeling frustrated due to the apparent absence of a solution for his symptoms, Participant A expressed to Professor Hume his desire to get involved with brain health research. Participant A not only wanted answers to his own questions, but also wanted to benefit others who may be going through a similar situation.

Professor Hume informed Participant A of an upcoming sabbatical research visit from GRHRP co-principal investigator Professor Patrick Neary from the University of Regina in Canada. Professor Hume informed Participant A that the purpose of Professor Neary’s sabbatical was to share and establish his Neary protocol for brain health assessment in New Zealand. The protocol, which has been published in separate components [7–12], enables the neurophysiological assessment of an athletes’ history of SRC and current brain health. Given Participant A’s self-reported background, he was advised he would qualify as a participant for testing using the Neary protocol. Participant A enthusiastically agreed to participate and was scheduled to be the first New Zealand participant to be assessed using the Neary protocol. AUT ethics #18/45 was gained to enable Participant A to be a case study to evaluate if the neurophysiological assessment battery could detect impaired functions suspected to underlie persistent SRC symptoms.

## Protocol

Athletes who experience persistent symptoms after SRC typically experience these symptoms as a result of one or more underlying impairments in normal physiological, vestibulo-ocular, and/or cervical spine function as a result of the initial SRC [13, 14]. In more rare cases, persistent complaints may be due to neuropathological changes, excitoimmunotoxicity, and/or in rare instances by chronic traumatic encephalopathy [1, 15–17]. The majority of persistent symptoms occur as a consequence of physiological dysfunction following SRC [18]. The Neary protocol is a battery of tests (requiring approximately 60 min to administer) that assess neurophysiological responses under a variety of conditions including the following: in a resting seated position; during changes in posture; during a cognitively and visually demanding ‘Where’s Wally’ task; while undergoing repeated 20-s breath holds; and during a 15-min cycle exercise test with workload increases every 5 min. During this testing protocol measures of heart rate variability, cerebrovascular reactivity, and respiratory gas exchange are collected in parallel throughout the duration of the protocol [7–9]. The Neary protocol is sensitive to changes in these neurophysiological functions following acute SRC, allowing for the identification of atypical physiological responses likely underlying symptoms subjectively reported by concussed athletes [7–9]. The GRHRP has implemented the Neary protocol in Canada and the UK from May to December 2017 to investigate if differences in neurophysiological responses could be observed between retired rugby players and athletes from non-contact sports with no history of SRC.

Participant A underwent the first Neary protocol testing in New Zealand at SPRINZ located within AUT Millennium on 7 March 2018. Participant A also had a second testing session on March 20 2018 to ascertain if any changes in his neurophysiological responses were observed between assessments.

Recent recommendations from Ellis et al. [6, 14] suggested that isolated dysfunction of the vestibular, ocular, and/or cervical spine neurological sub-system may be the cause of PCS when impaired neurophysiological function is not apparent. The results of Participant A’s Neary protocol assessments did not reveal any notable impairments in neurophysiological functions. indicating the possibility that his symptoms could be a product of vestibulo-ocular and/or cervicogenic dysfunction secondary to SRC as described by Ellis et al. [6]. To further investigate what might be causing Participant A’s symptoms, he was scheduled for a medical consultation at a dedicated concussion clinic. Assessments included:

- Clinical examination (consisting of a thorough medical and concussion history),
- Physical examination (involving neurological assessment and signs of autonomic dysfunction, cervical spine examination),
- Vestibular screening (using vestibulo-ocular motor screening tools),
- Neurocognitive assessment (using SCAT 5 testing),
- Balance and gait assessment (using Balance Error Scoring System and Tandem Gait 3 m Walk time), and
- Treadmill exercise testing (using a modified Balke protocol as per the Buffalo Concussion Treadmill Test) [19].

Participant A performed within normative data limits on all assessment tests. During the medical history component of the consultation, Participant A reported unilateral tinnitus on the right that arose after pistol shooting approximately 8 years ago. All of Participant A’s SRCs took place before the age of 30, and Participant A reported that symptoms of these SRCs resolved within days or weeks after the injury. Participant A’s current symptoms began when he was 33 years old. The symptoms recalled from his previous SRCs did not present with similarities to his current symptom complaints.

The conclusion was that the current symptoms appeared to be consistent with a peripheral vestibular cause rather than persistent complications of SRC, due to the lack of a temporal relationship between the onset of his symptoms and the previous concussions. Participant A was therefore referred for medical review with a neurologist. The neurologist ordered an updated structural MRI scan and conducted a further neurological assessment. Both the MRI and neurological assessment were normal (except for a minor note of fluid trapped in the inner ear). Participant A exhibited normal neck movements, carotid artery responses, reflexes, tone, strength, coordination, and a negative Dix-Hallpike test. Participant A did not present with typical features of Meniere’s disease or benign positional vertigo. Participant A’s reports of imbalance and intermittent dizziness appeared to be related to the right inner ear and the neurologist noted that these results were suggestive of disembarkment syndrome and that peripheral vestibular disturbance was the likely cause. Participant A was referred for further assessment and management at a specialised dizziness and balance centre where he underwent assessments administered by both a vestibular therapist and vestibular audiologist. The specialists noted normal dynamic visual acuity. However, unilateral tinnitus and an asymmetric hearing profile in combination with otolithic dysfunction as the main finding of the assessment were confirmed during a gait assessment wherein large head movements reproduced some of Participant A’s symptoms. This response to head movements during walking indicated stress on the otolithic organ was responsible for increased symptomology. Accordingly, Participant A received an exercise programme to increase demand on the otolith to promote adaptation to improve, and hopefully permanently resolve, the cause of his symptoms. Follow-up appointments at the dizziness and balance centre were planned to progress and review the effect of the exercise programme every 3–4 weeks.

## Discussion

Current consensus indicates that the majority of individuals with SRC will experience complete symptom resolution within 10–14 days of their injury [1]. However, little is known about the long-term effects of SRC, and estimates suggest between 20 and 40% of individuals will experience persistent complications for weeks or months following SRC [4, 20]. A major challenge when working with individuals who have had a history of SRC is the comorbidity shared between symptoms of SRC and an extensive array of other conditions such as migraine, depression, mental health disorders, learning disabilities, sleep disorders, or (in Participant A’s case) peripheral vestibular dysfunction and tinnitus [6, 21]. Further increasing this challenge is the ‘invisible’ nature of SRC and these other comorbid conditions, meaning these conditions are unlike a broken bone or a soft tissue injury that can easily be confirmed or ruled out using validated clinical tests, X-ray, or MRI. This is especially evident in cases where an individual who has a history of SRC is reporting persistent issues for months or years since their most recent SRC. How is the patient or the clinician supposed to know if the reported persistent symptoms are due to SRC, a comorbid condition sharing symptoms with SRC, or a combination of the two? To date, differentiating between symptoms of SRC and other comorbid conditions relies heavily on the level of experience and expertise of the medical professional(s) assessing these athletes. While media attention surrounding the long-term effects of SRC led to the series of events resulting in Participant A’s eventual diagnosis, we feel as though it is within reason to suggest his peripheral vestibular dysfunction and otolithic dysfunction were unrelated to his history of SRC based on the findings of his clinical evaluations in addition to temporal relationship between his last SRC and the onset of his symptoms. However, this cannot be concluded with absolute certainty due to the current limited understanding of long-term consequences associated with SRC.

In the past, SRCs were not considered serious injuries, therefore were not assessed and monitored thoroughly by medical professionals throughout the athlete’s recovery. The absence of monitoring might increase the risk of secondary injury and potential long-term deficits and complications for an athlete [1, 3, 4, 15, 16]. Largely due to improved media reporting of scientific studies, and case studies of current and retired players having sustained concussion, there is increased awareness of the danger of SRC. Athletes may no longer be underestimating the severity of SRC injury. However, fear of persistent symptoms associated with SRC such as memory issues, headaches, and dizziness may lead to athletes with a history of SRC (and clinicians treating these athletes) to inappropriately conclude all symptoms or impairments are manifestations due to concussion. These preconceptions and premature conclusions before a thorough interdisciplinary assessment can be conducted could be detrimental to the long-term health of the athlete by missing the true cause of reported symptoms. The findings of Participant A’s case suggest that a comprehensive and systematic collaborative effort by an interdisciplinary team may be essential to determine the cause of persistent symptom reports in current or retired athletes with a history of SRC. There is no individual testing procedure that can determine the origin of persistent SRC or non-SRC symptoms; rather, an exhaustive process of elimination is necessary to rule out potential causes of symptoms until only the most logical and realistic cause(s) are left. This process of elimination approach is the only method at this point in time to identify and diagnose these types of ‘invisible’ pathologies. In New Zealand, the public healthcare model includes ACC, which allows injured patients to access necessary medical services for little to no personal financial cost. Even within this healthcare model, it took several weeks to coordinate appointments with all the medical professionals involved in the assessment and management of Participant A. In regions without public healthcare, individuals of low socio-economic status may struggle to afford and access the necessary services they require for their injury. Therefore, there is a need, both in New Zealand and globally, to develop and optimise interdisciplinary clinical models to streamline the management of patients with complex injuries such as SRC and related comorbidities. While this approach requires a substantial amount of communication and coordination between multiple healthcare disciplines, this effort is paramount to ensure proper management for individuals presenting with comorbid symptoms that may or may not be related to SRC. Once a final diagnosis has been made, then it is possible to design and administer an individualised treatment programme to address any underlying impairments causing the patient distress. Without this comprehensive and collaborative process, individuals may continue to suffer from their respective afflictions, potentially impacting their mental and social well-being as well as their ability to perform at school or work [1, 22]. Governing and medical organisations must embrace this challenge to ensure patients with serious injuries/conditions do not slip through the cracks of the healthcare system.

The introduction of the Neary protocol to New Zealand was a key component in the process of understanding the potential pathophysiological mechanism(s) responsible for Participant A’s symptoms. Participant A did not demonstrate any abnormalities in neurophysiological function during the physical elements of the Neary protocol. Additionally, Participant A did not struggle with the cognitively demanding ‘Where’s Wally’ component of the Neary protocol, nor did he report difficulties coping with cognitive load during his day-to-day life or at work. Participant A’s unremarkable findings from his two Neary protocol assessments were the first step in the identification of peripheral vestibular dysfunction and otolithic dysfunction responsible for his symptoms. Intolerance to physical and cognitive loading is indicative of a symptomatic SRC patient, and improvements in tolerance are used as clinical markers of recovery. In contrast, a symptomatic patient who exhibits normal tolerance to physical and cognitive loading indicates that referral for assessment by a specialised healthcare professional may be required to identify the source of symptomology [6, 23]. This may include referral to:

- A neurologist, for a comprehensive evaluation of the central and peripheral nervous systems;
- A psychologist, to screen for any mental health or mood disorders;
- A neuropsychologist, to gauge cognitive function and performance;
- And/or a vestibular therapist to assess central and peripheral vestibular systems.

Participant A’s history and early clinical evaluations did not indicate that referral to a neuropsychologist or a psychologist was necessary; however, this may not be the case for other individuals suffering from non-specific symptomology which may or may not be related to SRC. Therefore, stressing the need for individualised management on a case by case basis.

The GRHRP has three research clinics currently (Canada, UK, and now New Zealand) to collect Neary protocol data from retired rugby players with no history of SRC, retired rugby players with a history of multiple SRCs, and retired athletes who engaged in non-contact sports with no history of SRC. The aim of this international project is to determine if any differences in neurophysiological responses during the Neary protocol are present between these groups of athletes, and if differences are observed, do these differences relate to symptoms or impairments reported by the athletes. The GRHRP has preliminary pilot data collected in the UK to suggest that regional differences in the pre-frontal cortex exist between normal healthy control participants (without a history of SRC) and retired rugby players. Future findings from this GRHRP investigation into neurophysiological responses will enhance our current understanding of the long-term effects of playing rugby and/or a history of SRC on how the brain regulates physiological functions. While the Neary protocol was designed to detect changes in neurophysiological responses following SRC, the present case study suggests the Neary protocol may be an objective method to assist in discerning whether persistent issues reported by an individual with a history of SRC are due to SRC or potentially a comorbid cause, i.e. otolithic dysfunction. However, we provide a caveat that additional testing using blood pressure monitoring (which was not performed on Participant A) is necessary to confirm whether pressure alleviation occurred during the time of his testing sessions [9]. The potential discriminatory utility of the Neary protocol would require additional research. Nonetheless, improved objective screening protocols may help clinicians decide whether there were physiological contributions to reported symptoms. The overall knowledge gained from the on-going GRHRP project will benefit athletes, clinicians, and researchers by assisting in guiding the progression of future research and clinical practice.

## Conclusions

Lessons from Participant A’s case study show that on-going symptoms that patients may associate with the effects of concussions may not be related to concussion. In Participant A’s case, an eventual diagnosis of peripheral vestibular dysfunction and otolithic dysfunction was made. Increased awareness and changes in attitudes/policies in recent years have enabled a major leap forward in terms of protecting the short- and long-term health of athletes following SRC. Nevertheless, caution must be exercised when assessing and managing an individual with a history of SRC. This caution is necessary to confirm that all possibilities for contributing factors have been considered for SRC symptoms that are comorbid in nature with a variety of other conditions. Lack of a thorough assessment for athletes with a history of SRC who present with symptoms that are comorbid with conditions unrelated to SRC may result in missing the true cause of these symptoms. By missing the true contributing cause of symptoms, this may lead to the athlete experiencing prolonged issues for weeks, months, or years. To overcome these challenges, a clinical model involving the coordination and communication of a collaborative interdisciplinary team of experts is essential to ensure the patient receives the best and most appropriate care [6].

## Abbreviations

ACC: Accident Compensation Corporation
AUT: Auckland University of Technology
GRHRP: Global Rugby Health Research Programme
PCS: Persistent concussion symptoms
SPRINZ: Sports Performance Research Institute New Zealand
SRC: Sports-related concussion
